# Improving image-retrieval performance of foundation models in gastrointestinal endoscopic images

**DOI:** 10.3389/fmed.2025.1727884

**Published:** 2025-12-18

**Authors:** Kangsan Kim, Junseok Park, Sang Hyun Kim, Youngbae Hwang

**Affiliations:** 1 Department of Intelligent Systems and Robotics, Chungbuk National University, Cheongju, Republic of Korea; 2 Department of Internal Medicine, Soonchunhyang University College of Medicine, Seoul, Republic of Korea; 3 Department of Surgery, Soonchunhyang University College of Medicine, Seoul, Republic of Korea

**Keywords:** gastrointestinal endoscope, artificial intelligence, deep learning, image retrieval, foundation model

## Abstract

**Introduction:**

The quality of gastrointestinal endoscopy is verified by documenting specific required images, but identifying these images from the numerous photographs captured during a procedure is tedious. Conventional deep-learning approaches that aim to automate this process are often limited by subjective assessments and poor interpretability.

**Methods:**

We introduce a novel content-based image-retrieval framework that employs a dual-backbone architecture, integrating a general-purpose vision foundation model (DINOv2) and a domain-specific endoscopic model (GastroNet). The system is trained using parameter-efficient metric learning to generate discriminative embeddings for efficient similarity searches. The framework is evaluated on 3,500 public endoscopic images (from the Kvasir and HyperKvasir datasets) and validated on entirely unseen real-world and synthetic data.

**Results:**

Our model achieves state-of-the-art performance (97.71% Recall@1, 99.14% Recall@5, and 96.74% mean average precision), which is significantly superior to those of single-backbone baseline models. Ablation studies confirm that this improvement is primarily due to the two backbones capturing complementary features.

**Discussion:**

These findings demonstrate that the proposed dual-backbone framework offers an accurate and automated tool for assessing the procedural quality of gastrointestinal endoscopy and may facilitate more reliable quality control in clinical practice.

## 1 Introduction

Endoscopy is a critical diagnostic tool for gastrointestinal diseases, which enables the direct visual inspection of internal organs. For instance, during esophagogastroduodenoscopy (EGD), the entire stomach cannot be visualized in a single field of view; therefore, images of specific anatomical segments must be captured (1). These landmark images serve as crucial quality control indicators (2, 3). Currently, manually verifying these images from the numerous photographs taken is a tedious task, prompting the automation of the process (4, 5). However, traditional deep-learning-based approaches are hindered by the need for large, comprehensively annotated training datasets, and their susceptibility to overfitting limits their performance across diverse data (6–8).

As an alternative, content-based image retrieval (CBIR) offers a more flexible and interpretable paradigm for analyzing endoscopic images (9, 10). Instead of relying on predefined labels, CBIR operates by comparing the extracted features of a query image with those in a reference database to find similar items (11, 12). This approach enhances clinical explainability and better accommodates image variability; its performance can be further improved using supplementary techniques, such as triplet loss (13). Recent advancements in CBIR involve utilizing large-scale foundation models, which offer advantages such as reduced training requirements and improved generalizability (14).

Accordingly, this study proposes a framework that leverages a foundation model for endoscopic image retrieval. This system enables efficient search for similar images within endoscopic image datasets, guided by anatomically relevant features and contextual similarity to a user-defined reference image.

## 2 Materials and methods

**Figure 1:**
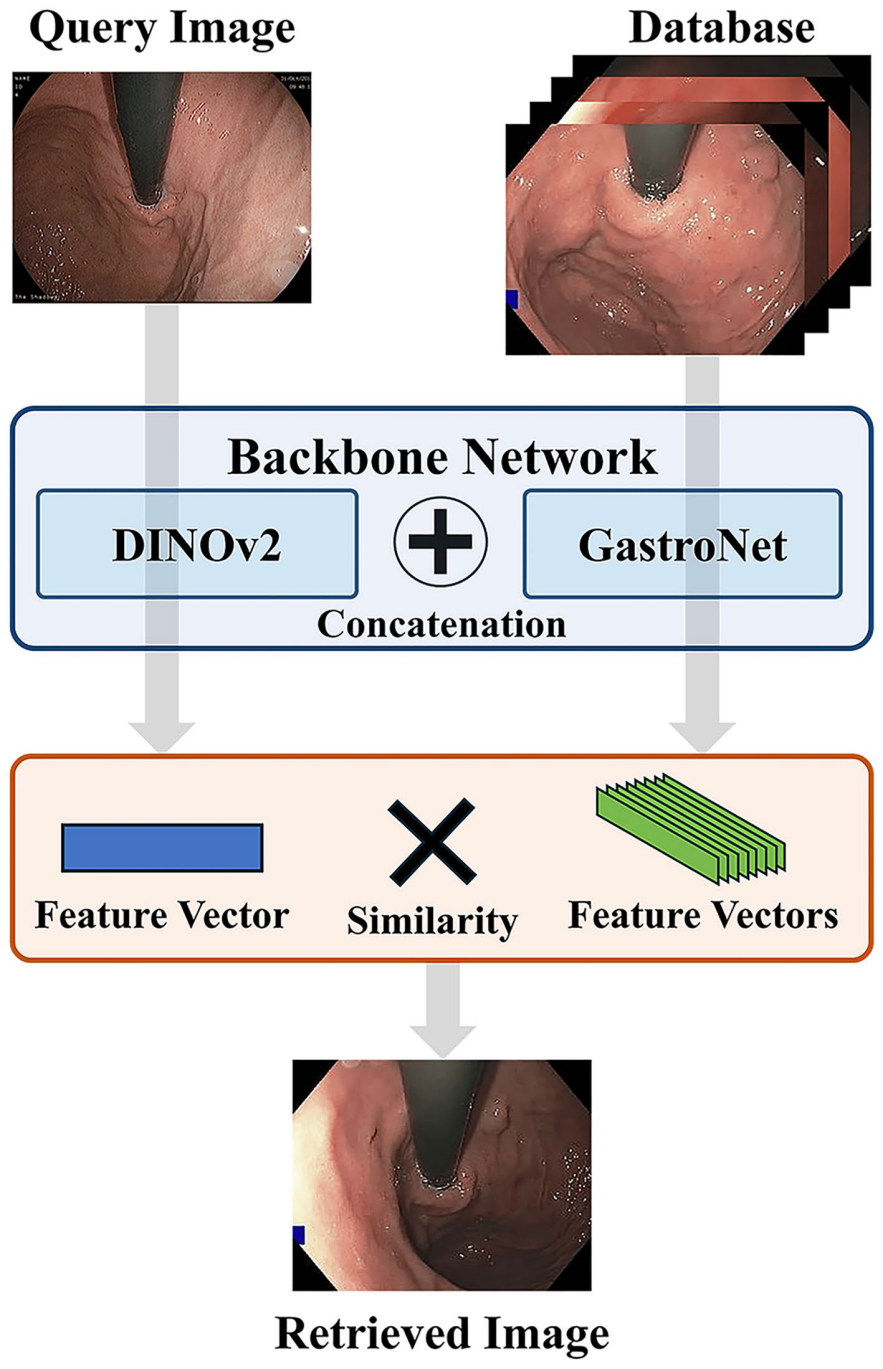
Flowchart of the proposed CBIR process. A query image and database images are passed through the dual-backbone network to generate feature vectors. The query vector is then compared against the database vectors using a similarity metric to retrieve the most visually similar image.

**Figure 2:**
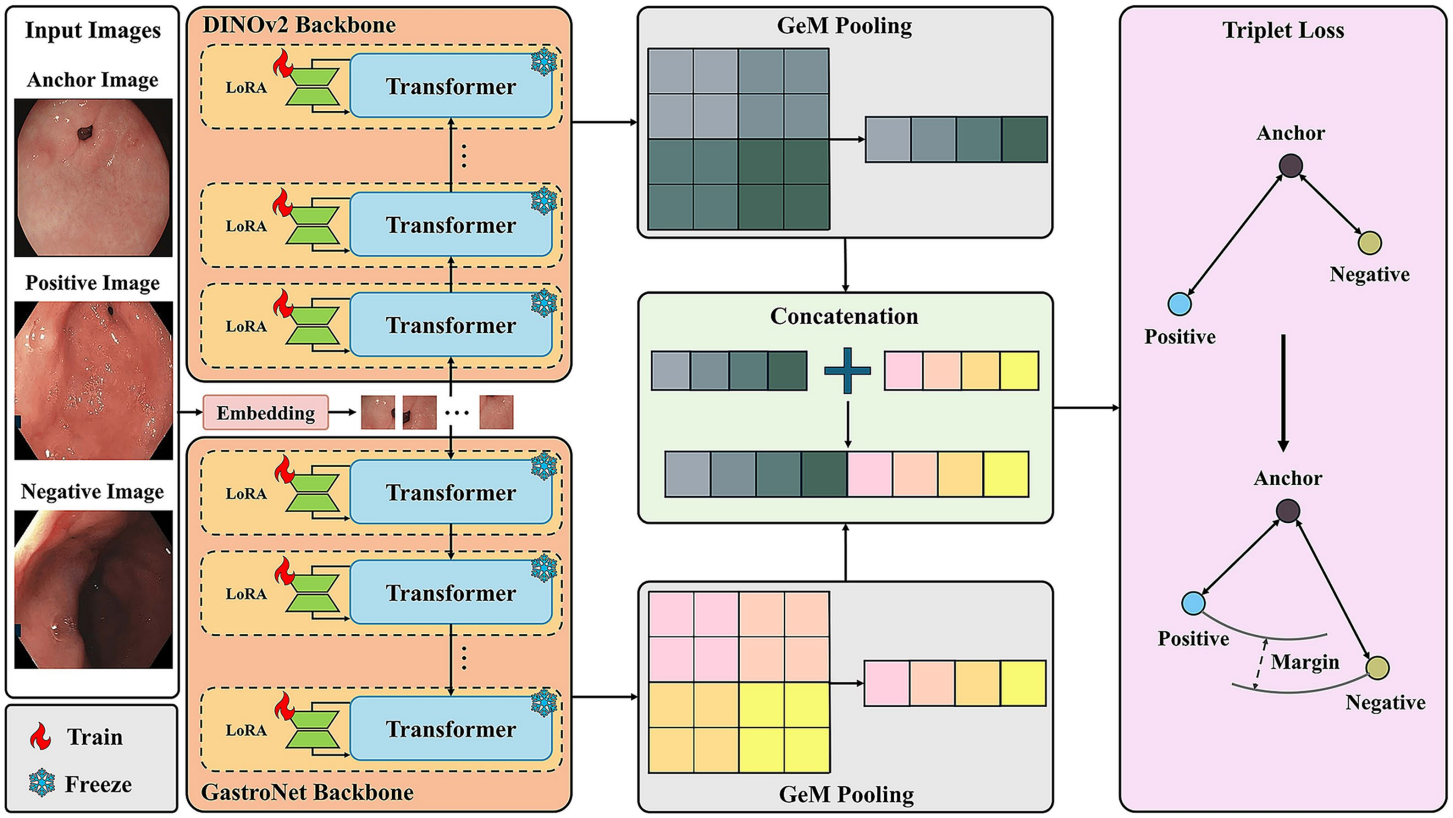
Architecture of the dual-backbone image-retrieval framework. Input images (anchor, positive, and negative) are processed by two parallel backbones: DINOv2 and GastroNet. PEFT is applied using LoRA modules, where pretrained transformer weights are frozen. Features from each backbone are pooled using generalized mean (GeM) pooling and then concatenated. The final embedding is optimized using a triplet loss function to minimize the distance between the anchor and positive while maximizing the distance to the negative.

The proposed CBIR system for gastrointestinal endoscopy images (Figure 1) processes a given query image by mapping it into a low-dimensional feature vector or embedding. It then retrieves the most visually similar images from a database by ranking their embeddings according to a distance metric, such as cosine similarity. The core feature of this framework is a novel dual-backbone feature extractor, whose architecture is detailed in Figure 2. This model synergistically combines representations from two distinct foundation models: DINOv2 (15) and GastroNet (16), which were pretrained on a broad corpus of natural images and on large-scale endoscopic data (five million images), respectively. To create a highly discriminative embedding space, the entire architecture was optimized using a triplet loss-based metric learning approach.

### 2.1 Dual-backbone-based feature extraction network

The premise of the dual-backbone architecture is that a general-purpose model, such as DINOv2 (15), captures fundamental visual primitives (e.g., shapes and textures), while a domain-specific model, such as GastroNet (16), extracts the fine-grained features unique to endoscopic environments. DINOv2 enables strong geometric and semantic generalization across visual domains, whereas GastroNet provides domain-specific sensitivity to mucosal texture and color variations. Hence, we adopted DINOv2 as the general-purpose backbone in this study. By fusing these complementary representations within a Vision Transformer (ViT) architecture (17), the framework generates a more discriminative embedding space than either model could produce alone, enabling robust anatomical similarity matching for endoscopic image retrieval.

### 2.2 Feature pooling and fusion

Following feature extraction, the multi-dimensional feature maps from the dual backbones undergo a pooling and fusion process, which is detailed in the central column of Figure 2. This stage is engineered to distill the rich information from both backbones into a compact, discriminative representation. Generalized mean (GeM) pooling is employed to transform the 2D feature maps from each backbone into a 1D vector (18). This method enhances the overall representational power of the embedding while preserving important local features. The GeM pooling operation is defined in Equation (1) as:


$$f_{k}^{\left(g\right)}={\left(\frac{1}{|X_{k}|}\sum_{x\in X_{k}}x^{p_{i}}\right)}^{\frac{1}{p_{k}}}$$ (1)

where the learnable parameter $p_{k}$ controls the pooling behavior. Setting $p_{k}=1$ is equivalent to average pooling, whereas $p_{k}\to \infty$ corresponds to max pooling.

To create a comprehensive feature vector that leverages the strengths of both models, the 1D vectors from each backbone were fused via concatenation, as illustrated in Figure 2. This concatenated embedding simultaneously captures general-purpose visual structures and domain-specific endoscopic details in a single, unified representation. While attention-based or learnable weighting mechanisms can also enable adaptive feature fusion, we adopted simple concatenation to ensure architectural simplicity and training stability. This approach preserves complementary information from both backbones without introducing additional parameters or alignment constraints, offering an efficient and widely applicable baseline for multimodal fusion (19, 20).

### 2.3 Model training

The similarity between two feature vectors is measured using cosine distance, which offers superior robustness to other metrics. The distance between two vectors, a and b , is calculated in Equation (2) as follows:


$$\text{dist}(a,b)=1-\frac{a\cdot b}{∥a∥∥b∥}$$ (2)

The embedding space was optimized for the retrieval task using a triplet loss function, which operates on data triplets comprising an anchor ( $x_{a}$ ), a positive ( $x_{p}$ , an image similar to the anchor), and a negative ( $x_{n}$ , an image dissimilar to the anchor). The objective is to ensure that the anchor-positive distance is smaller than the anchor-negative distance by at least a predefined margin, α . This process guides the model to form distinct clusters of similar images within the embedding space, thereby optimizing its structure for retrieval. The loss is formally defined in Equation (3) as


$$\begin{array}{c} L_{\text{triplet}}=\sum_{i=1}^{N}\max(d(f(x_{a}^{i}),f(x_{p}^{i}))-d(f(x_{a}^{i}),f(x_{n}^{i}))+\alpha ,0) \end{array}$$ (3)

where f(x) is the embedding generated by our dual-backbone model for an input image x , and d(,) is the cosine distance from Equation 2.

As illustrated in Figure 2, we adopted a parameter-efficient fine-tuning (PEFT) strategy using low-rank adaptation (LoRA) to minimize computational demands and the risk of overfitting (19–21).

### 2.4 Datasets

To validate model performance, we used four distinct datasets for training, in-domain evaluation, generalization testing, and synthetic querying. For model training and in-domain evaluation, we used two public datasets—Kvasir and HyperKvasir (22, 23)—which contain a substantial collection of endoscopic images categorized by anatomical landmarks and pathological findings. An endoscopic expert selected seven relevant classes, excluding cases involving artificial dyeing agents or ambiguous interpretations: esophagitis, normal pylorus, normal Z-line, ulcerative colitis, normal cecum, polyps, and retroflex stomach (Table 1). A balanced training dataset of 3,500 images was created by randomly sampling 500 images from each class. For the test set, 200 images were randomly sampled from the remaining images in each class.

**Table 1:** Distribution of images from the Kvasir and HyperKvasir datasets across the seven selected classes.

Dataset	Esophagitis	Normal pylorus	Normal Z-line	Ulcerative colitis	Normal cecum	Polyps	Retroflex stomach
Kvasir	1,000	1,000	1,000	1,000	1,000	1,000	—
HyperKvasir	663	999	932	851	1,009	1,028	764

To test the model with synthetic queries virtually generated at Soonchunhyang University, we used a dataset created by applying synthetic textures—derived from real endoscopic videos—to a 3D model constructed from CT scans. These images were used exclusively as queries to assess performance on out-of-distribution data.

Finally, to assess generalizability to unseen clinical data, we employed the GastroHUN dataset (24), which contains an extensive collection of clinical endoscopic videos with anatomical labels. We created a comprehensive search database by sampling frames from these videos and used it to validate the robustness and clinical applicability of our model.

### 2.5 Evaluation metrics and parameter settings

Two standard retrieval metrics were adopted to evaluate model performance: Recall@k and mean average precision (mAP) (25–27). Recall@k measures the proportion of queries for which at least one correct image is retrieved within the top-k results. We adopted Recall@1 (R@1) to assess the models’ ability to immediately find a relevant match and Recall@5 (R@5) to evaluate performance in a practical scenario where a clinician might review the top few suggestions.

mAP provides a more holistic evaluation of the ranked retrieval results. Unlike Recall@k, it considers the rank of all correct images in the retrieved list, rewarding models that place correct items higher and penalizing those that place correct items lower. When calculated over all queries, it provides a comprehensive, single-figure summary of a model’s overall retrieval quality.

All models were implemented using the PyTorch framework and trained on a workstation with a single NVIDIA RTX 3090 GPU (24 GB VRAM). We employed the AdamW optimizer, a robust variant of Adam that improves regularization by decoupling weight decay from the gradient update (28). A learning rate of $1\times 10^{-5}$ was used, as this is a standard choice that facilitates stable convergence when fine-tuning large pretrained models. The model was trained for 30 epochs. The margin hyperparameter *α* in the triplet loss function was set to 0.3, a value selected to ensure sufficient separation between dissimilar classes without making the training excessively difficult.

## 3 Results

### 3.1 Image-retrieval results

To evaluate the effectiveness of our proposed model, we conducted comparative experiments against multiple representative baseline architectures. These baselines were organized into three categories: (1) commonly used convolutional neural network architectures pretrained on ImageNet (29) [ResNet50 (30), VGG19 (31), DenseNet (32), and SENet (33)]; (2) supervised Transformer models [ViT-L/16 (17) and Swin Transformer (34)]; and (3) the foundation models that are direct components of our architecture [DINOv1 (35), DINOv2 (15), and GastroNet (16)]. For a fair and rigorous comparison, all baseline models were fine-tuned on our training set under identical experimental conditions. The quantitative results for the Kvasir and HyperKvasir test sets are presented in Table 2.

**Table 2:** Image-retrieval performance comparison of various models on the Kvasir and HyperKvasir test sets.

Model	Published in	Recall@1 (%)	Recall@5 (%)	mAP (%)
ResNet50	CVPR 2016	76.57	92.86	51.33
VGG19	ICLR 2015	75.71	92.57	49.17
DenseNet	CVPR 2017	80.86	90.23	51.77
SENet	CVPR 2018	81.14	91.38	51.48
ViT	ICLR 2021	82.86	93.35	45.07
Swin Transformer	ICCV 2021	81.71	91.46	53.75
DINOv1	ICCV 2021	83.71	93.29	60.36
DINOv2	TMLR 2023	86.86	93.43	71.73
GastroNet	MIA 2024	90.57	94.57	83.19
Ours		**97.71**	**99.14**	**96.74**

mAP, mean average precision; CVPR, Computer Vision and Pattern Recognition; ICLR, International Conference on Learning Representations; ICCV, International Conference on Computer Vision; TMLR, Transactions on Machine Learning Research; MIA, Medical Image Analysis. Bold values indicate the performance of the proposed dual-backbone model (Ours).

**Figure 3:**
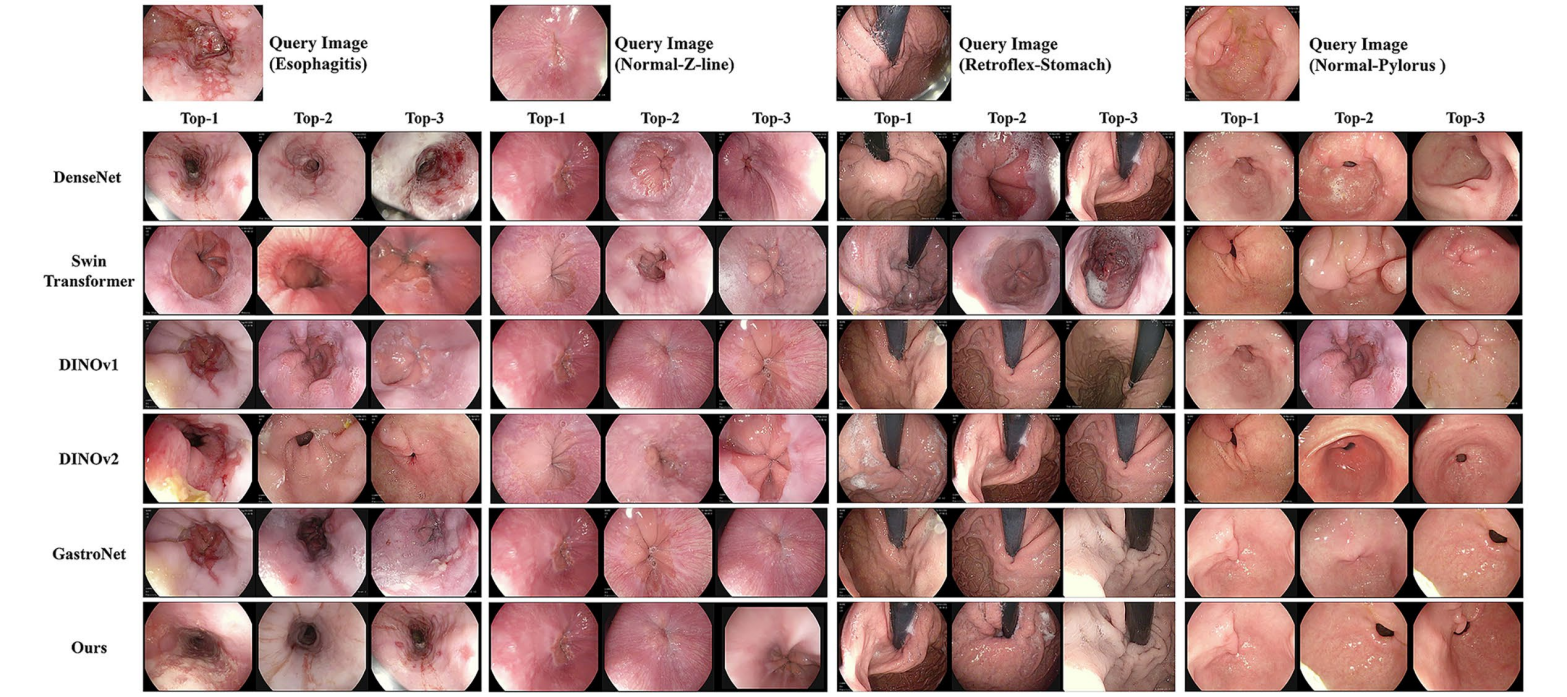
Qualitative comparison of image-retrieval results from the proposed model and representative baselines. The four columns show the retrieval results for four different query images: esophagitis, normal Z-line, retroflex-stomach, and normal-pylorus. The rows display the top-three most similar images retrieved by each model, allowing for a visual assessment of their performance in identifying relevant anatomical landmarks and pathological conditions.

Our model achieved state-of-the-art performance across all metrics, attaining a Recall@1 of 97.71%, Recall@5 of 99.14%, and mAP of 96.74%. This performance significantly surpasses all baselines, including the performance of the strongest single-backbone model, GastroNet (Recall@1 = 90.57%, mAP = 83.19%). Notably, our dual-backbone fusion strategy delivers a 7.14 percentage point improvement in Recall@1 over GastroNet and 13.55% improvement in mAP. Figure 3 presents a qualitative comparison of retrieval results for four representative examples. The figure, which compares our model’s top-three retrievals against those of key baseline models, visually confirms the superior quality of our model in consistently identifying semantically and visually coherent results. For esophagitis queries, our model retrieves images that match the inflammatory patterns and severity, whereas baselines such as the Swin Transformer incorrectly retrieve images of healthy esophageal tissue. Similarly, for viewpoint-sensitive queries such as normal pylorus, our model correctly identifies the precise endoscopic orientation (e.g., distal pyloric view). By contrast, even strong baselines such as DINOv1 retrieve the correct organ but fail to match the required perspective. These qualitative results corroborate the quantitative data presented in Table 2, demonstrating the robustness of our dual-backbone architecture.

A detailed class-wise evaluation is presented in Table 3, further highlighting the model’s robust performance. The model demonstrates exceptional accuracy in identifying anatomical landmarks, achieving a Recall@1 of over 98% and Recall@5 of 100% for normal-pylorus, normal-Z-line, retroflex-stomach classes. The mAP was also high, particularly for the normal pylorus (99.99%), normal Z-line (98.46%), and retroflex stomach (100%) classes, while remaining strong for pathological findings such as Esophagitis (89.76%).

**Table 3:** Class-wise image-retrieval performance on the Kvasir and HyperKvasir test set.

Class	DINOv2			GastroNet			Ours		
	Recall@1 (%)	Recall@5 (%)	mAP (%)	Recall@1 (%)	Recall@5 (%)	mAP (%)	Recall@1 (%)	Recall@5 (%)	mAP (%)
Esophagitis	84.00	90.00	69.85	88.00	90.00	79.84	**96.00**	**98.00**	**89.76**
Normal pylorus	92.00	98.00	76.32	92.00	100.00	86.16	**100.00**	**100.00**	**99.99**
Normal Z-line	86.00	96.00	68.48	90.00	96.00	83.47	**98.00**	**100.00**	**98.46**
Retroflex stomach	94.00	98.00	93.38	94.00	100.00	96.71	**100.00**	**100.00**	**100.00**
Ulcerative colitis	78.00	88.00	61.47	90.00	92.00	76.64	**96.00**	**98.00**	**93.80**
Normal cecum	88.00	90.00	71.67	88.00	90.00	78.92	**96.00**	**98.00**	**96.88**
Polyps	86.00	94.00	60.94	92.00	94.00	80.62	**98.00**	**100.00**	**98.30**

mAP, mean average precision. Bold values indicate the performance of the proposed dual-backbone model (Ours).

**Figure 4:**
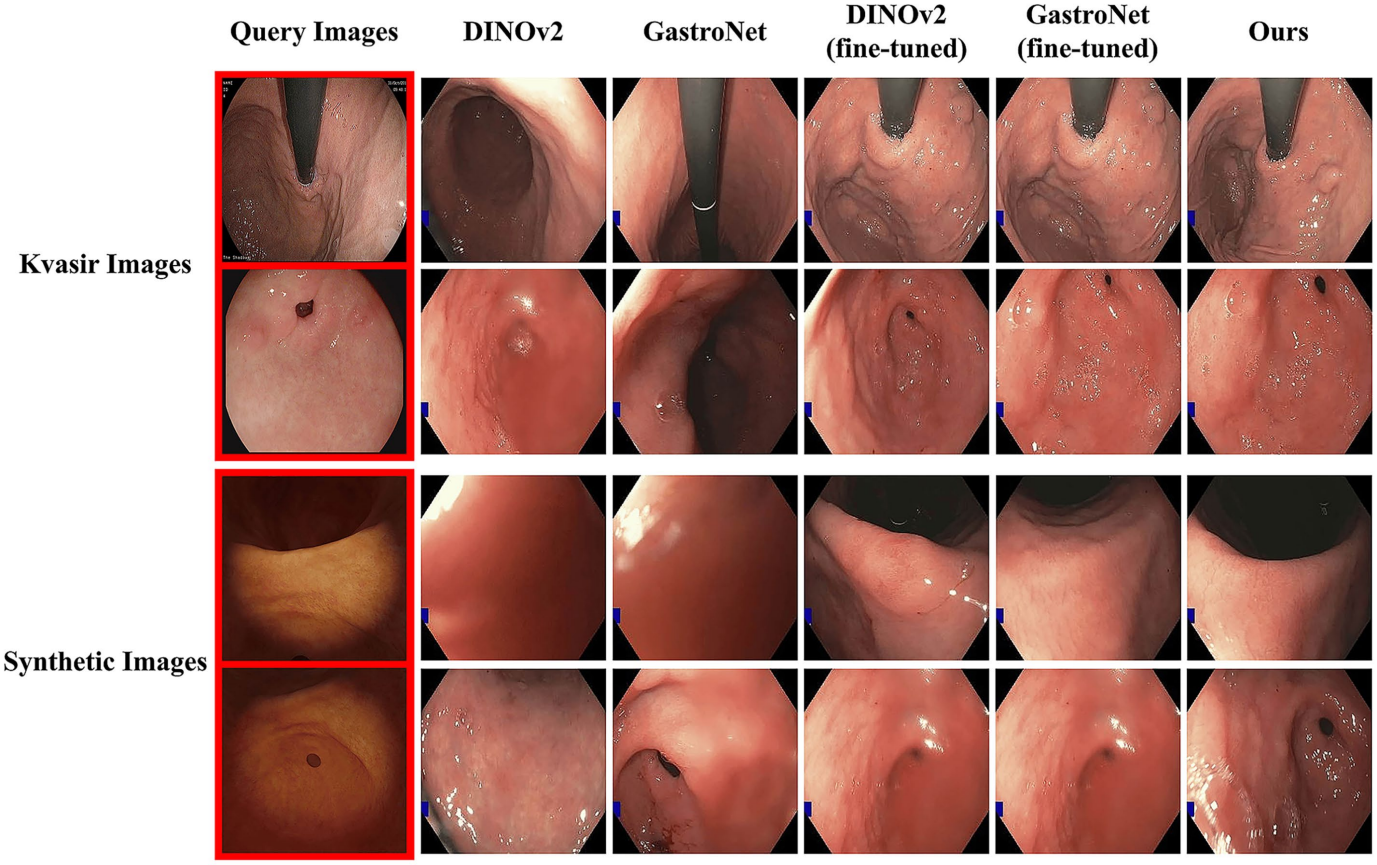
Qualitative comparison of zero-shot generalization performance. The images on the first column are selected as query images from the Kvasir dataset (top two rows) and a synthetic dataset (bottom two rows). The rightmost columns display the top-retrieved image from five different model configurations.

Additionally, we evaluated zero-shot generalization by using the unseen GastroHUN dataset as the search database, with queries drawn from both the public Kvasir/HyperKvasir datasets and a proprietary synthetic dataset. This configuration rigorously assessed the model’s capacity to bridge the domain gap between diverse query sources and a real-world clinical database. As shown in Figure 4, this evaluation yielded three key findings. First, the pretrained GastroNet significantly outperforms DINOv2, highlighting the benefit of endoscopic-specific pretraining. Second, triplet loss fine-tuning substantially improves the performance of all models. Finally and most critically, our dual-backbone architecture consistently outperforms all other configurations by retrieving semantically precise matches, demonstrating its superior robustness and generalizability.

To further examine the trade-off between retrieval accuracy and computational efficiency, we measured the average inference time per image on both GPU and CPU for the representative models (DINOv2, GastroNet, and ours). As summarized in Table 4, our dual-backbone model achieved the highest retrieval accuracy with moderate computational overhead (15.47 ms on GPU and 58.2 ms on CPU). Compared with the fastest single-backbone model (GastroNet, 10.91 ms on GPU and 26.5 ms on CPU), our model shows approximately 1.4 × higher GPU latency and 2.2 × higher CPU latency, reflecting the additional computation from dual-branch fusion. Nevertheless, the performance gain (Recall@1, 97.71% vs. 90.57%) demonstrates a favorable balance between accuracy and efficiency. Latency was measured on an NVIDIA RTX 3090 (24 GB VRAM) and an Intel Core i7-10700F CPU (2.90 GHz, 8 cores, 16 threads).

**Table 4:** GPU and CPU inference latency comparison of representative models (DINOv2, GastroNet, and our model).

Model	Recall@1 (%)	Recall@5 (%)	mAP (%)	GPU latency (ms)	CPU latency (ms)
DINOv2	86.86	93.43	71.73	10.60	34.2
GastroNet	90.57	94.57	83.19	10.91	26.5
Our model	97.71	99.14	96.74	15.47	58.2

mAP, mean average precision.

### 3.2 Ablation study

**Figure 5:**
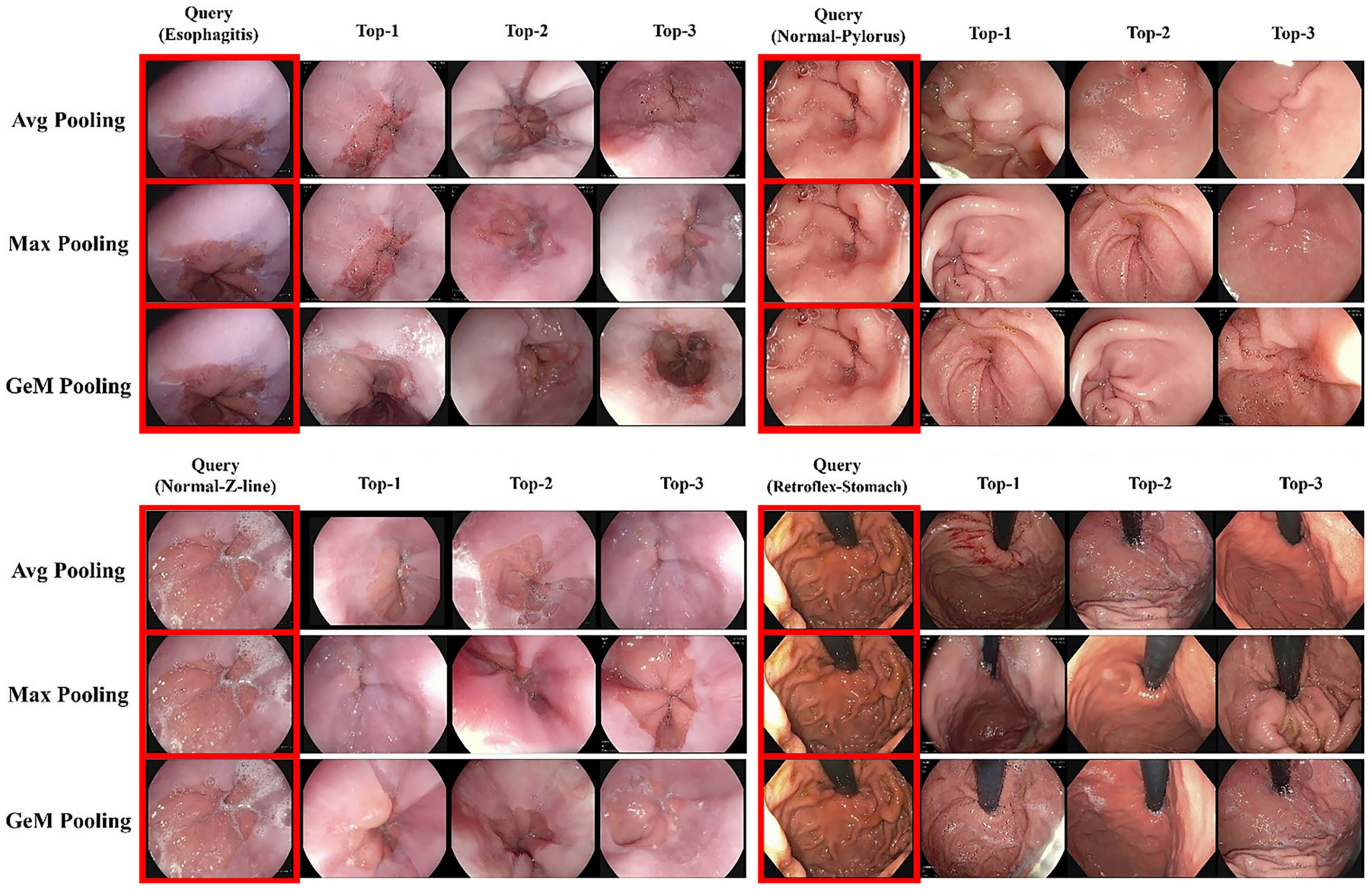
Qualitative comparison of retrieval performance with different pooling methods. The rows display the top-three retrieved images using average, max, and GeM pooling. The columns correspond to four different query images (esophagitis, normal pylorus, normal Z-line, and retroflex-stomach). The visual results demonstrate the superior performance of GeM pooling in retrieving visually consistent and semantically relevant images compared to the other methods.

An ablation study was conducted to isolate the contribution of each component, and the results are summarized in Table 5. The dual-backbone fusion (GastroNet + DINOv2) achieved an mAP of 96.74%, outperforming both the single-backbone GastroNet (96.26%) and DINOv2 (76.57%), thereby confirming the synergistic effect of combining domain-specific and general features. Furthermore, GeM pooling (96.74% mAP) significantly outperformed average (84.05%) and max (89.75%) pooling. As shown in Figure 5, GeM pooling demonstrated superior visual coherence in its retrievals. Finally, triplet loss (96.74% mAP) outperformed contrastive loss (91.58%), validating its efficacy in structuring the embedding space for fine-grained retrieval.

**Table 5:** Ablation study on the effect of dual-backbone fusion.

DINOv2	GastroNet	Fusion	Average pooling	Max pooling	GeM pooling	Contrastive loss	Triplet loss	Recall@1 (%)	Recall@5 (%)	mAP (%)
✓					✓		✓	87.71	94.43	76.57
	✓				✓		✓	91.14	95.71	96.26
		✓	✓				✓	90.57	96.63	84.05
		✓		✓			✓	93.15	96.29	89.75
		✓			✓	✓		95.71	96.29	91.58
		✓			✓		✓	**97.71**	**99.14**	**96.74**

mAP, mean average precision. Bold values indicate the performance of the proposed dual-backbone model (Ours).

### 3.3 Clinical effectiveness of the dual-backbone image retrieval model

**Figure 6:**
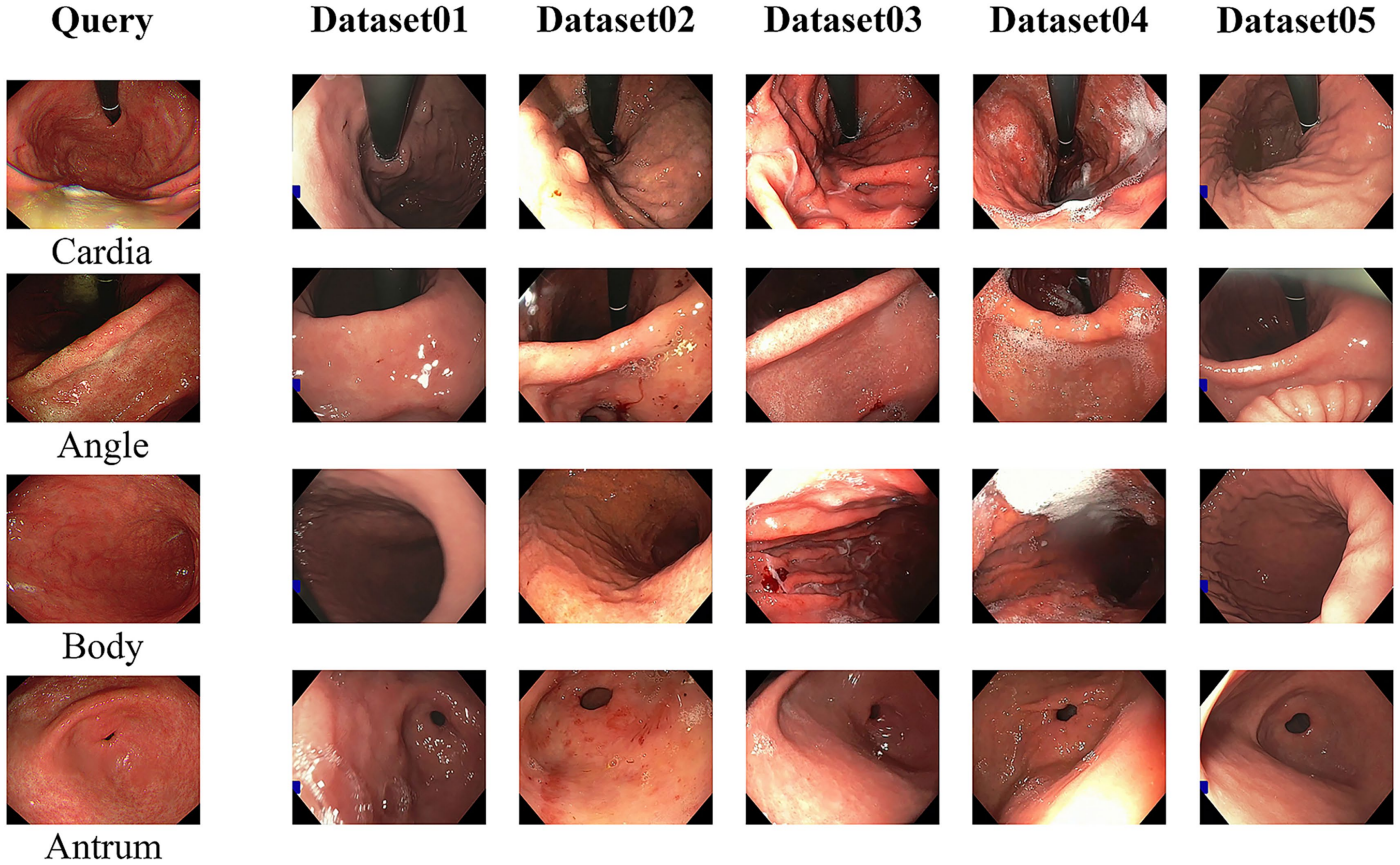
Image retrieval results for the recommended stomach observation sites. Using the four recommended stomach observation site images as queries, our dual-backbone-based model successfully extracted images from the GastroHUN video dataset at a clinically satisfactory level.

To verify the effective operation of our dual-backbone image retrieval model across diverse clinical endoscopy videos, we conducted a query using actual esophagogastroduodenoscopy videos that accurately captured the four anatomical sites of stomach recommended by clinical guidelines: cardia, angle, body, and antrum (1). For each query, we extracted the top-one retrieved frame from the GastroHUN dataset videos and had the results reviewed by a clinical endoscopy specialist to confirm its accuracy. Our model successfully retrieved the correct images for all four targeted observation sites. The results from five representative videos are shown in Figure 6.

## 4 Discussion

Accurate photo documentation is a critical aspect of gastrointestinal endoscopy and serves as a significant quality indicator for examinations (2, 3). During EGD in particular, capturing images of essential observation sites is strongly emphasized (1). In Korea, medical institutions evaluate the standard management of gastrointestinal endoscopy by verifying whether photographs of the recommended observation sites are correctly documented in randomly selected cases (36). However, manually selecting and verifying these images is extremely labor-intensive. The application of deep learning to automate this process has yielded impressive results. For instance, Choi et al. (4) introduced a multiclass classification system to recognize eight landmarks in the pictorial results of EGD, achieving an accuracy of 97.58%. Their study used 2,599 images captured from 250 participants using a specific Olympus CV-290 endoscope system. Similarly, Ahn et al. (5) developed an automated tool to capture 11 landmark images from endoscopic videos, achieving 98.16% accuracy; for their model, 102,798 photos from 3,309 examinations were used for training and validation. Despite these excellent results, their performance cannot be assured across various endoscopic models because the training data were derived from a limited range of hardware systems (8). Furthermore, the need for such a large, expertly annotated dataset complicates the development and scalability of these deep-learning models.

Our dual-backbone model was designed to overcome the limitations of these methods, and it demonstrated superior image-retrieval performance. This efficacy was confirmed not only quantitatively on a modest public dataset of 3,500 images but also through its robust performance on entirely unseen real-world and synthetic data. The architecture is built on the core hypothesis that synergy can be achieved by combining two distinct models: a general-purpose model that captures fundamental visual primitives (e.g., shapes, textures, and spatial relationships) and a domain-specific model that discerns fine-grained features unique to the endoscopic environment. To achieve this, we used DINOv2, which was pretrained on a large-scale natural image dataset using a self-supervised learning scheme, to provide a foundation understanding of visual scenes and ensure robustness. This was integrated with its domain-specific counterpart, GastroNet, a foundation model pretrained on a large dataset of approximately 5 million endoscopic images. Both backbones leverage the ViT architecture (17), processing images by partitioning them into sequences of fixed-size patches and projecting them into high-dimensional embeddings. The fusion of these complementary representations ultimately yielded a more robust and discriminative embedding than either model could achieve independently. This dual-backbone approach aligns with recent research highlighting the importance of domain-specific feature learning for abnormality detection and the integration of self-supervised pretrained foundations with domain-specific models for mitigating data scarcity and improving robustness to clinical variability in medical imaging (37, 38).

To further analyze this synergistic interaction, we conducted an additional experiment to examine how different training strategies affect feature alignment between the two backbones. Specifically, we implemented a staged training strategy in which the GastroNet branch was frozen while only the DINOv2 branch was trained, followed by the joint fine-tuning of both backbones. We then tested two frozen-stage settings—7 epochs (mAP 95.86%, Recall@1 97.43%) and 10 epochs (mAP 96.58%, Recall@1 97.71%)—and found that both yielded slightly worse results than the dual-backbone baseline (mAP 96.74%, Recall@1 97.71%). These results indicate that simultaneous co-optimization enables more stable and efficient feature alignment between the two backbones compared with sequential adaptation. Therefore, we retained the joint fine-tuning strategy as the final configuration for all experiments.

**Figure 7:**
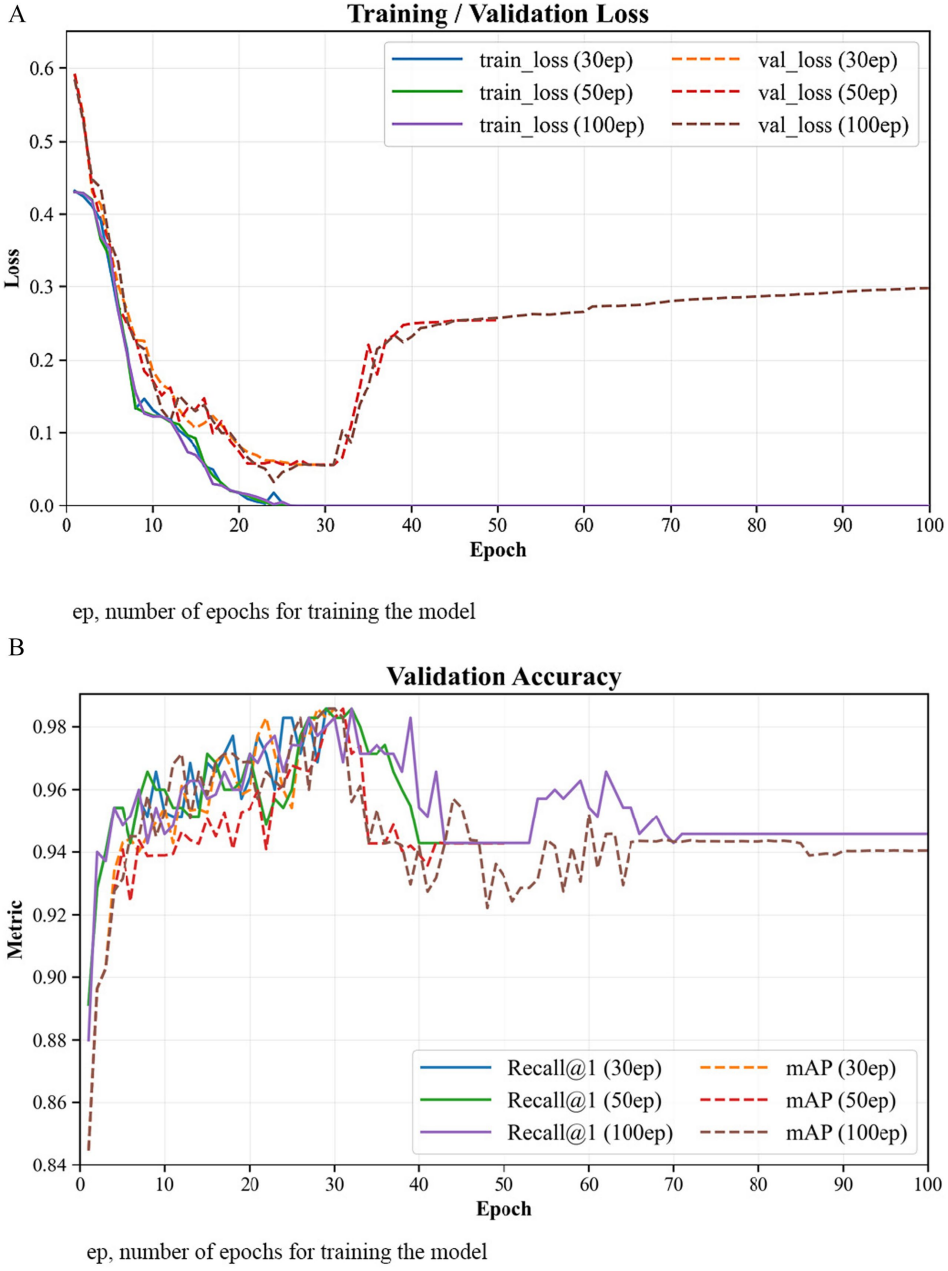
Training and validation curves across different epoch settings (30, 50, and 100). Both **(A)** training and validation loss and **(B)** validation accuracy (Recall@1 and mAP) curves stabilize around the 25th–30th epoch, after which extensive training leads to overfitting, confirming that 30 epochs are sufficient for convergence.

To verify whether the model was sufficiently trained, we examined the training and validation loss, as well as validation accuracy (Recall@1 and mAP), across different epoch settings (30, 50, and 100 epochs). As shown in Figure 7, both loss and accuracy curves stabilized around the 25th to 30th epoch. The validation metrics peaked near 30 epochs, after which extended training caused a gradual rise in validation loss and a slight decrease in accuracy, indicating overfitting. Fluctuations in validation metrics were also observed, likely due to the inherent instability of triplet-based embedding learning under semi-hard negative sampling (39). Therefore, the optimal training duration for all final experiments was set as 30 epochs.

CBIR has emerged as a flexible and interpretable paradigm that overcomes the constraints of traditional deep-learning-based classification (10). Instead of assigning rigid labels, a CBIR system retrieves visually similar images from a reference database, an approach that allows for direct visual comparison, enhances clinical explainability, and accommodates images that defy predefined categories. Modern CBIR pipelines integrate three core components: feature extraction, similarity search, and metric learning. The process begins with feature extraction, where an image’s visual content is converted into a quantitative vector representation, known as an embedding (40). Next, a similarity search compares the query embedding against the database; for large-scale applications, this is often accelerated using approximate nearest neighbor algorithms, such as Facebook AI Similarity Search, to avoid computationally prohibitive exhaustive searches (41). Finally, metric learning techniques, such as triplet loss (13), optimize the quality of the embedding space by refining it to pull similar images closer together while pushing dissimilar ones apart. This integrated pipeline provides a scalable and interpretable framework uniquely suited to the complexity of endoscopic imaging. Our model’s training process and architecture were specifically tailored to the challenges of endoscopic imaging. Using a metric learning approach, triplets of anchor, positive, and negative images were input into the pretrained ViT-based backbones to generate embedding vectors (17). The ViT architecture, which partitions each image into patches before transformation, is adept at capturing global context. We then employed GeM pooling (18), a critical component for this task. As a generalized form of both average and max pooling, GeM pooling excels at preserving local features while enhancing the embedding’s overall representational power. This capability proved particularly beneficial for endoscopic images, which are often characterized by subtle anatomical differences and complex textures, ultimately enabling the extraction of highly discriminative embeddings.

Our work aligns the recent paradigm shift in CBIR from traditional task-specific fine-tuning toward leveraging large-scale foundation models. However, directly fine-tuning these massive models is computationally demanding and prone to overfitting. To mitigate these challenges, we employed a PEFT strategy using LoRA (21). This technique involves freezing the pretrained model weights and inserting small, trainable LoRA modules into each transformer block. By representing weight updates with low-rank matrices, LoRA drastically reduces the number of trainable parameters, i.e., from 43.68 million for full fine-tuning to just 0.615 million in our implementation. This approach yields significant advantages: it improves computational efficiency, reduces operational costs, and prevents catastrophic forgetting by preserving the model’s pretrained knowledge while skillfully adapting it to our retrieval task (42). Ultimately, PEFT strategies like LoRA enable the practical adaptation of powerful foundation models for specialized tasks with minimal trainable parameters.

While foundation models offer robust representations that excel in few-shot and zero-shot scenarios (14), their direct application to medical data faces significant challenges. A major challenge is the pronounced domain shift between the natural images used for pretraining and the unique visual characteristics of medical data, which can degrade performance and necessitate sophisticated adaptation techniques (43, 44). Development is further constrained by strict privacy regulations and the high cost of expert annotation for data curation (45). Moreover, the inherent opacity of these “black box” algorithms can impede clinical adoption, where transparency is essential for building trust and ensuring patient safety (46). In light of these challenges, we acknowledge the limitations of our study. Although our model demonstrated strong performance, its robustness must be further validated on larger, multi-center datasets to confirm its generalizability across different clinical environments.

**Figure 8:**
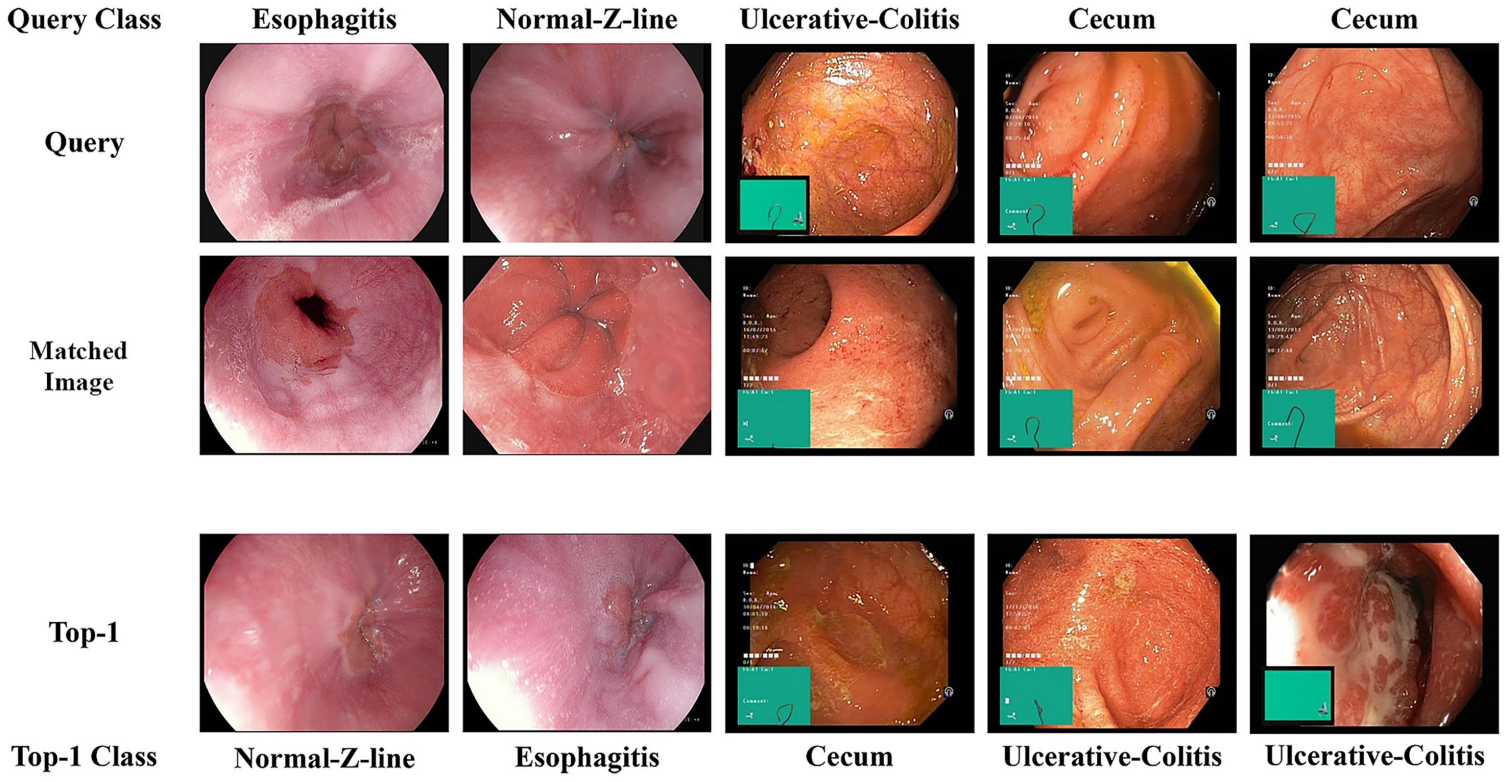
Qualitative error analysis of representative failure cases. Each column shows a query, one example image of matched class, and the top-one retrieval result. Most errors occurred between visually similar classes in the same organ, mainly due to subtle variations in viewpoint or illumination.

To contextualize these challenges, we analyzed representative retrieval failures. The qualitative results are shown in Figure 8. Most retrieval errors occurred between visually similar or anatomically adjacent categories (e.g., normal Z-line and esophagitis) and were primarily driven by subtle variations in viewpoint or illumination rather than gross semantic confusion. These observations highlight the need for stronger fine-grained discrimination under variable imaging conditions. To further address these limitations, future work should extend the single-image retrieval framework to multi-image and multimodal settings, incorporating CLIP-based vision–language alignment to enhance semantic robustness (47, 48). Furthermore, more advanced and adaptive fusion strategies, such as attention-based or cross-modal mechanisms, should be investigated to further enhance feature interaction and retrieval robustness. Future work should also explore model compression and quantization to ensure its efficient deployment in real-time settings with constrained computational resources.

Although our dual-backbone architecture slightly increases inference latency (15.47 ms vs. 10.91 ms on GPU, ≈ 1.42×), this trade-off is considered acceptable given the substantial improvement in retrieval accuracy (+7.1% in Recall@1 and +13.6% in mAP). The proposed dual-backbone model therefore achieves a favorable balance between computational efficiency and accuracy. Additionally, incorporating an image quality assessment module could further enhance dataset consistency and improve the robustness and reliability of retrieval performance, which we plan to implement in future work.

In conclusion, we introduced a dual-backbone retrieval framework that establishes a new state-of-the-art for the automated quality control of endoscopic documentation. Our work demonstrates that the synergistic combination of a general-purpose and a domain-specific model yields a representation more powerful than either could achieve independently. By leveraging this architecture alongside triplet-loss-based metric learning, our approach surpasses traditional classification methods, offering superior explainability and the flexibility to manage ambiguous or novel visual data. Overall, this research contributes to the development of more effective and clinically applicable AI technologies for medical imaging.

The full implementation of the proposed framework is available at https://github.com/Girin325/ImageRetrieval-with-DualModel.

## Data Availability

The datasets presented in this article are not readily available because the images can be reconstructed, thereby compromising the privacy of the patients. Requests to access the datasets should be directed to JP, junspark@schmc.ac.kr.
